# Description of 3 Cases in Vietnam of Aspirin Desensitization in Patients With Coronary Artery Disease and Coexisting Aspirin Hypersensitivity

**DOI:** 10.1097/WOX.0b013e3182718327

**Published:** 2012-11-15

**Authors:** Dinh Van Nguyen, Hieu Chu Chi, Doan Van Nguyen, Timothy J Craig, Sheryl van Nunen

**Affiliations:** 1 Allergy Department, Hanoi Medical University, Hanoi, Vietnam; 2 Center of Allergology and Clinical Immunology, Bach Mai Hospital, Hanoi, Vietnam; 3 Section of Allergy, Asthma and Immunology, Pennsylvania State University, Hershey, PA; 4 Sydney Medical School-Northern, University of Sydney, Sydney, Australia; 5 Department of Clinical Immunology and Allergy, Royal North Shore Hospital, Sydney, Australia

**Keywords:** Vietnam, aspirin (ASA) hypersensitivity, ASA desensitization, coronary artery disease

## Abstract

Aspirin (ASA) hypersensitivity comprises types I to III (Cox-1 mediated) and types IV and V (IgE antibody mediated). Rapid, low-dose (81-325 mg/day) ASA desensitization regimens are known to be useful in establishing ASA tolerance in patients with coronary artery disease and coexisting ASA/nonsteroidal anti-inflammatory drug hypersensitivity. We document 3 cases in Vietnam of desensitization to ASA in patients with coronary artery disease and coexisting ASA hypersensitivity. One of these 3 patients had probable immune-mediated hypersensitivity, whereas the remaining 2 had probable Cox-1-mediated reactions. The regimen of desensitization we employed for each patient was designed to account for the probable mechanism of hypersensitivity in the individual and further modified according to the degree of tolerance observed, with all 3 patients eventually achieving a daily cardioprotective dosage of ASA.

## 

In 1971, British pharmacologist John Vane demonstrated that aspirin [acetylsalicylic acid (ASA)], an inhibitor of platelet activation and aggregation, irreversibly inhibits the platelet-dependent enzyme cyclooxygenase (COX), thereby preventing the synthesis of prostaglandins. Subsequent researchers identified 2 COX isoenzymes: COX-1 and COX-2 [1,2]. In platelets, the COX-1 enzyme produces thromboxane A2, a powerful promoter of platelet aggregation. Thus, aspirin, by irreversibly inactivating COX-1, blocks the generation of thromboxane A2, thereby exerting its antiplatelet effect. The role of ASA in the primary and secondary prevention of cardiovascular events has been established recently and treatment with anti-platelet agents including ASA can lead to a 31% even reduction rate [3]. However, some patients are unable to tolerate acetylsalicylic acid (aspirin) due to hypersensitivity.

ASA hypersensitivity comprises 5 distinct types, types I to III (Cox-1 mediated) and types IV and V type (IgE antibody mediated), with the classification based on the mechanism of hypersensitivity, risk factors, and cross sensitivity to nonsteroidal anti-inflammatory drugs (NSAIDs). Type I hyper-sensitivity manifests as respiratory symptoms such as rhinitis and asthma. Type II is observed in patients with chronic idiopathic urticaria and manifests as angioedema and an exacerbation of the urticaria. Urticaria and angioedema occurs in type III hypersensitivity without chronic idiopathic urticaria, with these symptoms occurring after the first dose of aspirin or NSAID. Types IV and V hypersensitivities, on the other hand, are mediated by aspirin-directed immunoglobulin E antibodies that rarely cross-react with other NSAIDs. Patients with type IV reactions present with angioedema and urticaria, whereas type V reactions are typical of anaphylaxis [4].

The incidence of ASA hypersensitivity ranges in the general population from 0.6% to 2.5%, and in adults with asthma, it ranges from 4.3% to 11%[5,6]; however, the prevalence of ASA hypersensitivity among individuals with coronary artery disease (CAD) in the Asia-Pacific region is not known precisely [7]. Notwithstanding, low-dose (81-325 mg/d), rapid ASA desensitization regimens appear to be useful in patients from countries other than Vietnam in the Asia-Pacific region, who have CAD and coexisting ASA/NSAID hyper-sensitivity, including those who have undergone percutaneous coronary intervention [7].

Our report documents 3 cases in Vietnam of desensitization to ASA in patients with CAD and coexisting aspirin hypersensitivity. One of these 3 patients had probable immune-mediated hypersensitivity, whereas the remaining 2 had probable COX-1-mediated reactions. Several protocols for ASA desensitization have been published, and the protocols have been summarized [4,8].

In our series, we chose to employ a regimen of desensitization for each patient that was designed or selected by us to account for both the probable mechanism of hypersensitivity operative in the individual and their clinical state. These slower protocols were then further modified according to the degree of tolerance observed. All 3 patients eventually achieved a cardioprotective dosage of aspirin delivered in a single daily dose.

## Case Reports

### Case 1

#### History

A 43-year-old Vietnamese man, referred to the Center of Allergology and Clinical Immunology, Bach Mai Hospital, Hanoi, Vietnam, had had asthma since childhood and had previously experienced episodes of angioedema after the ingestion of minor, nonprescription analgesics (precise constituents unknown). He had been given 75 mg of clopidogrel and 300 mg of ASA before the planned insertion of percutaneous cardiac stents. Within 30 minutes thereafter, he had developed sneezing, periobital angioedema, and pharyngeal discomfort, which settled after antihistamine was administered.

#### Investigations

His spirometry was normal. Skin prick testing with ASA 1 mg/mL was positive. Total serum IgE was 106 U/L (normal range < 100 U/L). Ear, nose, and throat examination and nasal/sinus computed tomography scans were normal.

#### Results

In view of the likely immune-mediated mechanism operative in this patient and his compromised cardiac status, ASA desensitization began with 0.0001 mg of ASA. Initially, doses were doubled each 30 minutes, provided the peak expiratory flow was not reduced. Breakthrough symptoms, which settled with 180 mg of fexofenadine, occurred 10 minutes after a single dose of 50 mg and the protocol was further modified, by returning to lower doses which had been tolerated and decreasing the rate of dosage increase thereafter (see Table 1). By day 10, the patient tolerated 100 mg daily in a single dose, and he continues to do so.

**Table 1:** A Summary of the ASA Desensitization Regimens Used in Our 3 Patients

Case	Clinical Manifestations of ASA Hypersensitivity	Premedication	Initial ASA Dose (mg)*	Final ASA Dose (mg)	Initial Dosing Interval (minutes)	Time to Achieve a Single Daily Dose of 100 mg of ASA (days)	Breakthrough Reactions (on Maintenance Dosage After Final Protocol Given)	Follow-up (months)
1	Angioedema/urticaria	Fexofenadine	0.0001	100	30	10	No	8
2	Urticaria	Fexofenadine	10	100	90	6	No	6
3	Urticaria	Fexofenadine	10	100	90	6	No	3

*Initial dose of final protocol (milligram).

### Case 2

#### History

A 76-year-old Vietnamese man, with CAD requiring urgent stenting, presented to the Center of Allergology and Clinical Immunology, Bach Mai Hospital, Hanoi, Vietnam, with urticaria, which occurred only after aspirin ingestion. The onset of abdominal pain, nausea, and vomiting was noted to have occurred coincidentally with the suspected aspirin hypersensitivity.

#### Investigations

Spirometry and otolaryngological examination were normal. Total serum IgE was 1337 U/L (normal range < 100 U/L). *Ascaris lumbricoides *ova and parasites were identified in feces. Enzyme-linked immunosorbent assay for *Toxocara *and liver fluke was positive (1:3200 dilution).

#### Results

ASA desensitization was ceased after breakthrough symptoms at 100 mg; 400 mg of albendazole per day was given for 3 days. The protocol was then modified by starting at a lower dose and slowing the tempo of the dosage increases over a 6-day period. This patient now tolerates a daily dose of 100 mg of ASA (see Table 1).

### Case 3

#### History

Three years before his referral to the Center of Allergology and Clinical Immunology, Bach Mai Hospital, Hanoi, Vietnam, a 66-year-old Vietnamese man had been prescribed ASA for stable angina. However, ASA had been ceased 1 year before the presentation due to supervening ASA hypersensitivity manifest as urticaria. In addition, he had a history of a transient ischemic attack 2 years ago and childhood asthma. On this occasion, he had been admitted with a myocardial infarct, prompting consideration of ASA desensitization.

#### Investigations

Total serum IgE was 570 U/L (normal range < 100 U/L). *Ascaris lumbricoides *ova and parasites were negative in feces, and enzyme-linked immunosorbent assay for *Toxocara *and liver fluke was negative. There was no abnormality on ear, nose, and throat examination or thoracic examination.

#### Results

Initial desensitization with ASA was complicated by urticaria occurring 2 hours after the last dose of 100 mg. The protocol was then modified by starting at a lower dose and slowing the tempo of the dosage increases over a 6-day period. This patient now tolerates a daily dose of 100 mg of ASA (see Table 1).

## Discussion

In this report, we have documented 3 cases in Vietnam of desensitization to ASA in patients with CAD and coexisting aspirin hypersensitivity. Although the precise prevalence of ASA hypersensitivity remains to be determined in Vietnam, our report indicates that the condition exists in the Vietnamese population in both immune-mediated types and the COX-1-mediated types of reactivity. The patient described in case 1, with a skin test positive at 1 mg/mL of ASA and angioedema and urticaria after ASA, appears most likely to have type IV hypersensitivity. On the other hand, the patients described in cases 2 and 3, with urticaria occurring only after ASA ingestion, have a type III, COX-1-mediated reaction pattern. We are unable to comment upon whether the full range of hypersensitivity types occur in the Vietnamese due to the small numbers in this initial series; however, clinical experience with patients other than those described here indicates that types I, II, and V ASA hypersensitivity do occur in the Vietnamese.

Several protocols for ASA desensitization, identified by MEDLINE search, have been published between 1996 and 2006, and these were reviewed by Page and Schroeder in 2007 [8]. Rapid, low-dose (81-325 mg/d) ASA desensitization regimens are known to be useful in establishing ASA tolerance in patients with CAD and coexisting ASA/NSAID hyper-sensitivity. These protocols are invariably useful as a starting point for ASA desensitization; however, in our experience, to achieve desensitization in an individual patient, modification may be necessary, and ab initio consideration of both the probable mechanism of hypersensitivity operative in the individual and their clinical state is helpful in designing or selecting a regimen of desensitization for each patient. Thus, in view of the likely immune-mediated mechanism operative in the patient described as case 1 and his compromised cardiac status, ASA desensitization began with 0.0001 mg of ASA and doses were doubled each 30 minutes. However, in the patients described in cases 2 and 3, with probable COX-1-mediated hypersensitivity, initial doses of 10 mg were used. Arguably, the need for modification may have been due to our reliance upon fexofenadine alone as pretreatment in our patients, as some other investigators, Wong et al [9] and Silberman et al [10], have employed montelukast and/or oral corticosteroids as pretreatment medications in their desensitization regimens. Rossini et al [11], Hobbs and Lyle [12] and Dalmau et al [13], however, used no pretreatment medications and achieved similar success to Wong et al [9] and Silberman et al [10] [pooled success rate for protocols utilizing pretreatment regimens 87.5% (21 of 24 subjects) and pooled success rate for protocols not using pretreatment regimens 89.4% (42 of 47 subjects)].

Although all 3 patients achieved a daily cardioprotective dosage of aspirin, breakthrough symptoms, which settled after the administration of antihistamine (additional 180 mg of fexofenadine), necessitated modification of the regimen in all 3 cases. In case 1, urticaria occurred 10 minutes after a single dose of 50 mg. Thereafter, the protocol was modified, by returning to lower doses that had been tolerated and decreasing the rate of dosage increase thereafter, with a daily dose of 100 mg in a single dose achieved by day 10 and a combined daily dose of at least 100 mg being achieved from day 2. Intervals between doses of 30 minutes were thought appropriate for this patient, given the immediate reaction he demonstrated. In cases 2 and 3, breakthrough symptoms of urticaria occurred 2 to 3 hours after the first dose of 100 mg given during the desensitization. Modification of the regimen, returning to lower doses which had been tolerated, increasing the interval between doses (to 1.5 hours), and decreasing the rate of dosage increase thereafter over a 6-day regimen, was successful, with a daily dose of 100 mg in a single dose achieved by day 6, with a daily dose of 100 mg achieved from day 2 onward, in both cases. In case 2, ASA desensitization had been ceased when breakthrough symptoms occurred, and albendazole was administered for parasitism before the ASA desensitization was resumed, in case helminthic infestation was present, and it was promoting the occurrence of the urticaria. It is interesting to speculate that the difficulty in achieving tolerance to a single daily dose of 100 mg in the patients described as cases 2 and 3 may relate to their underlying parasitism, which may have aggravated their tendency to produce urticarial lesions [14].

## Conclusions

We conclude that both immune-mediated and COX-1-mediated ASA hypersensitivity occur in Vietnamese patients and that desensitization with ASA is successful in these individuals, albeit with appropriate modification of the ASA desensitization protocols both ab initio, according to the probable mechanism of hypersensitivity operative and the clinical state of the patient, and during desensitization, according to the clinical progress of the patient.
